# Longitudinal evaluation of Tau‐P301L transgenic mice reveals no cognitive impairments at 17 months of age

**DOI:** 10.1002/brb3.896

**Published:** 2017-12-18

**Authors:** Brianne A. Kent, Christopher J. Heath, Chi Hun Kim, Rosemary Ahrens, Paul E. Fraser, Peter St George‐Hyslop, Timothy J. Bussey, Lisa M. Saksida

**Affiliations:** ^1^ Department of Medicine University of British Columbia Vancouver BC Canada; ^2^ Department of Life, Health and Chemical Sciences The Open University Milton Keynes UK; ^3^ Department of Psychiatry University of Oxford Oxford UK; ^4^ Tanz Centre for Research in Neurodegenerative Diseases University of Toronto Toronto ON Canada; ^5^ Cambridge Institute for Medical Research University of Cambridge Cambridge UK; ^6^ Department of Psychology and MRC & Wellcome Trust Behavioural and Clinical Neuroscience Institute University of Cambridge Cambridge UK; ^7^ Molecular Medicine Research Group, Robarts Research Institute & Department of Physiology and Pharmacology, Schulich School of Medicine & Dentistry Western University London ON Canada; ^8^ The Brain and Mind Institute Western University London ON Canada

**Keywords:** Alzheimer's disease, attention, frontotemporal dementia, P301L, recognition memory, tau

## Abstract

**Introduction:**

Tau is a microtubule‐associated binding protein implicated in neurodegenerative tauopathies, including frontotemporal dementia (FTD) and Alzheimer's disease (AD). These diseases result in the intracellular accumulation of hyperphosphorylated tau in the form of neurofibrillary tangles, the presence of which is associated with cognitive deficits.

**Methods:**

We conducted a longitudinal behavioral study to provide a profile of the TgTau(P301L)23027 transgenic mouse in multiple cognitive domains across multiple ages. P301L is the tau mutation most frequently observed in patients with frontotemporal dementia with parkinsonism linked to chromosome 17 (FTDP‐17) and this mouse model recapitulates the progressive development of glial and neurofibrillary tangles, and associated cerebral atrophy observed in patients. We examined frontal cortex‐dependent executive function and attention with the touchscreen 5‐choice serial reaction time test (5‐CSRTT) and assessed the function of temporal cortical structures using novel object recognition (OR).

**Results:**

Despite using sensitive tasks, there were no apparent changes in executive function, attention, or recognition memory in the transgenic mice from 5 to 17 months of age.

**Conclusions:**

This study represents the first comprehensive longitudinal analysis of cognition in the TgTau^P301L^ mouse model and suggests that this model is not ideal for studying early attention and recognition memory impairments associated with tauopathy. However, spatial and object recognition memory impairments were observed during follow‐up assessments when the mice were 18 and 21 months, respectively. These impairments are consistent with previous publications, and with a dementia‐like phenotype in these mice when aged.

## 1 INTRODUCTION

Alzheimer's disease (AD) and frontotemporal dementia (FTD) belong to a class of neurodegenerative disorders referred to as tauopathies. Tauopathies are histologically characterized by abnormal intracellular accumulation of hyperphosphorylated tau. Encoded by the MAPT gene, tau is a microtubule‐associated binding phosphoprotein involved in the assembly and stabilization of the cytoskeleton, which regulates neuronal processes and axonal transport. During pathogenesis of tauopathies, brain dysfunction and degeneration are linked to the progressive accumulation of hyperphosphorylated tau aggregates that form intracellular, filamentous inclusions, and neurofibrillary tangles (NFT; see Wang, Xia, Grundke‐Iqbal, & Iqbal, 2013 for a review). Patients diagnosed with tauopathies often experience impairments in multiple mnemonic and nonmnemonic cognitive domains, such as attention and executive control.

In human patients, abnormal tau aggregates are observed in brain regions exhibiting neuronal loss, suggesting that dysregulation of tau may cause the neuronal cell death associated with the disease pathology (Gomez‐Isla et al., 1997; Spires‐Jones, Stoothoff, de Calignon, Jones, & Hyman, 2009). Both NFTs and smaller tau oligomers are associated with neurotoxicity and cognitive deficits (see Ren & Sahara, 2013 for a review), and abnormal tau can contribute to neuronal dysfunction independently and prior to NFTs forming (Berger et al., 2007; Rocher et al., 2010; Santacruz et al., 2005; Wittmann, 2001). For example, a mouse model expressing a repressible mutant form of tau showed improved memory and less neuronal cell loss when tau expression was suppressed, even though NFTs remained unaffected (Santacruz et al., 2005).

The intracellular accumulation of tau aggregates also parallels memory disturbances and AD diagnosis criteria (Braak & Braak, 1995; Ohm, Muller, Braak, & Bohl, 1995). Because it may take up to 40 years from the first appearance of NFTs for a clinical diagnosis of AD, there is great interest in the role of tau in the earliest cellular changes that lead to functional deficits (Ohm et al., 1995).

Patients with tauopathies, such as AD and FTD, show central executive functioning impairment, demonstrating compromised performance on tasks assessing working memory, attention, and executive control (Nedjam, Devouche, & Barba, 2004; Stopford, Thompson, Neary, Richardson, & Snowden, 2012). The specific strains of misfolded tau species generated in each disorder selectively affect distinct brain regions, which are vulnerable to different forms of inclusions (Clavaguera, Akatsu, et al., 2013; Clavaguera, Lavenir, et al., 2013; Sanders et al., 2014). For example, FTD patients with parkinsonism linked to chromosome 17 (FTDP‐17) show severe atrophy in the frontotemporal lobe, varying degrees of neurodegeneration in subcortical nuclei, and tau‐positive pretangles, neurofibrillary tangles, and glial fibrillary tangles (Foster et al., 1997). However, the precise clinical and histological profile of FTDP‐17 is dependent on the specific MAPT mutation expressed by an individual patient. A number of mutations in the MAPT gene have been associated with FTDP‐17. Among these, the P301L mutation in MAPT exon 10 that results in a Pro→ Leu change at amino acid 301 (Bird et al., 1999; Dumanchin et al., 1998; Hutton et al., 1998; Nasreddine et al., 1999; Rizzu, Van Swieten, Joosse, & Hasegawa, 1999) is most frequently observed in patients with FTDP‐17 (Poorkaj et al., 2001).

Transgenic animal models that exhibit tau pathology are important for developing effective therapeutics. Failings in developing effective treatments result in part from our incomplete understanding of the causal mechanisms underlying disease progression and the difficulty in recapitulating dementia in animal models, which impedes translation to the clinic. The transgenic (TgTau^P301L^) mice expressing the P301L mutation within the longest form of tau (2N, 4R) have previously been shown to exhibit tau pathology development in the hippocampus, amygdala, and cerebral cortex by 3 months of age, tau‐positive pretangles by 10 months of age, and extensive NFTs throughout the frontotemporal cortex at 18–24 months of age (Murakami et al., 2006). This progressive neuronal impairment and accumulation of NFT are associated with age‐related cognitive deficits, recapitulating the pathology seen in patients with FTD and AD (Murakami et al., 2006; Wakasaya et al., 2011).

To further examine the effects of P301L mutant tau, the aim of the following study was to provide a longitudinal assessment of the TgTau^P301L^ mouse model across three cognitive domains.

Firstly, the TgTau^P301L^ model was evaluated using the 5‐choice serial reaction time test (5‐CSRTT) to assess executive function and attention, because of the regional specificity of pathology in the TgTau^P301L^ model (i.e., frontotemporal cortex structures), and also because of the possible utility of these cognitive changes in early detection (Albert, Moss, Tanzi, & Jones, 2001; Baddeley, Baddeley, Bucks, & Wilcock, 2001; Collette, Van der Linden, & Salmon, 1999; Lawrence & Sahakian, 1995; Perry, Watson, & Hodges, 2000). Frontal cortex‐dependent executive function and attention were examined at 4, 7, 12, and 16 months of age using a touchscreen version of the 5‐CSRTT. Our laboratory has previously used this task successfully with the 3xTgAD and TgCRND8 models (Romberg, Horner, Bussey, & Saksida, 2013; Romberg, Mattson, Mughal, Bussey, & Saksida, 2011), and the task is similar to touchscreen‐based tasks used to study attention and executive functioning in patients (Sahakian, 1993; Sahakian & Coull, 1993).

Secondly, the TgTau^P301L^ model was evaluated using an object recognition task. Recognition memory represents a fundamental ability to identify an object and judge whether it has been previously encountered. Performance on visual recognition memory tasks is highly predictive of conversion to AD and impairments are considered by some to be an early cognitive biomarker of disease (Didic et al., 2013) and consistent with the extensive atrophy in medial temporal lobe structures associated with AD (Juottonen et al., 1998).

Object recognition memory was assessed in the same cohort of TgTau^P301L^ mice using the Decoupled version of the object recognition (OR) task at 5, 8, 13, and 17 months of age. The Decoupled variant of OR was developed by our laboratory group and has been used successfully to identify memory impairment in the TgCRND8 mouse model of AD (Romberg et al., 2012). The task allows us to differentiate between forgetting and false memory, which is important because even though patients diagnosed with AD exhibit profound memory deficits, they do not necessarily have accelerated rates of forgetting (Christensen, Kopelman, Stanhope, Lorentz, & Owen, 1998; Money, Kirk, & McNaughton, 1992).

Taken together, this study represents the first comprehensive longitudinal analysis of cognition in the TgTau^P301L^ mouse model. No deficits in executive function, attention, or object recognition memory were detected between 5 and 17 months of age. Because the tau pathology is slow to develop in this model, it is possible that compensatory changes masked some of the phenotypes (such as attentional impairment) seen in human patients. Follow‐up assessments using spatial memory tests at 18 and 21 months of age, however, were consistent with previous studies demonstrating impairments in the Morris Water Maze and radial arm maze between 9 and 13 months of age (Murakami et al., 2006).

## 2 METHODS

### 2.1 Animals

All experiments were performed in accordance with Canadian Council on Animal Care guidelines and UK Animals Scientific Procedures Act (1986 and the Amendment Regulations 2012) and approved by the Animal Care Committee at the University of Toronto and the Cambridge University local ethics committee. Nontransgenic and tau P301L transgenic mice were generated as previously described (Murakami et al., 2006). We studied twenty‐six male mice expressing a P301L mutant version of the longest form of human tau [denoted TgTau(P301L)23027, for brevity TgTau^P301L^] on the 129SvEvxFVB/N genetic background and non‐Tg littermates (Murakami et al., 2006). At the start of behavioral testing, mice (12 Tg^+^ and 14 Tg^−^) were 8–10 weeks of age. Only males were used in this study. Table 1 shows the sample sizes during each phase of testing.

**Table 1:** Timeline of testing and experimental design

Age (months)	Behavioral task	Sample size
0–2		
3–4	Pretraining	*N* = 26 (12 Tg^+^ and 14 Tg^−^)
5	5‐CSRTT	*N* = 25 (12 Tg^+^ and 13 Tg^−^)
6	Decoupled OR (1‐ and 24‐hr delay)	*N* = 24 (11 Tg^+^ and 13 Tg^−^)
7	5‐CSRTT	*N* = 24 (11 Tg^+^ and 13 Tg^−^)
8	Decoupled OR (1‐ and 24‐hr delay)	*N* = 23 (11 Tg^+^ and 12 Tg^−^)
12	5‐CSRTT	*N* = 23 (11 Tg^+^ and 12 Tg^−^)
13	Decoupled OR (1‐ and 24‐hr delay)	*N* = 23 (11 Tg^+^ and 12 Tg^−^)
16	5‐CSRTT	*N* = 22 (10 Tg^+^ and 12 Tg^−^)
17	Decoupled OR (1‐ and 24‐hr delay)	*N* = 22 (10 Tg^+^ and 12 Tg^−^)

To determine sample sizes for our study, power analyses were run on estimated effect sizes from previously published research (e.g., Romberg et al., 2011). For example, we previously demonstrated impaired performance on the 5‐CSRTT in the 3xTgAD mouse model of Alzheimer's disease (Romberg et al., 2011). In this study, sample sizes were *n* = 8 per genotype and estimated effect sizes were large, ranging from Cohen's *f* = 1.0–2.02. Using G*Power (power 0.8; α = .05), sample sizes needed to correctly detect effects of similar size would be *n* = 6–8 per group. We started with sample sizes larger than these estimates to enable us to detect smaller effects, potentially aiding early detection. Additionally, given our longitudinal design, we anticipated higher mortality rates at advanced ages and wanted to ensure large enough sample sizes to detect differences between genotypes once aged.

Mice were housed in groups of 2–3 on a 12‐hr light cycle (lights on 19:00–07:00). All behavioral testing was performed during lights off. Mice were provided with ad libitum access to water, but food was restricted prior to the start of behavioral testing to maintain body weight at 85%–90% of free‐feeding weight throughout the study. There were no differences between the weights of the Tg^+^ and Tg^−^ groups at any time point (*p* > .05).

### 2.2 Touchscreen 5‐choice serial reaction time test (5‐CSRTT)

Touchscreen 5‐CSRTT (Bartko et al., 2011; Romberg et al., 2011) was used to evaluate attention and executive function and was conducted as previously described (Horner et al., 2013; Mar et al., 2013). Briefly, mice were trained to respond to a white square stimulus on the screen using a 2‐s stimulus duration for a maximum of 40 trials or 60 min as the baseline measure. Once acquired, the subjects were assessed using a series of probe tests, in which stimulus duration, delay, and trials per session were systematically adjusted.

Mice were tested in sound‐ and light‐attenuating boxes with a ventilation system, house light, tone generator, and infrared light camera. The testing box enclosed a touchscreen operant chamber and reward delivery system (Campden Instruments Ltd., Loughborough, UK). Black plastic masks with five response windows were placed on the touchscreen to minimize unintended screen contact and to help focus attention. The system was controlled by Whisker and ABETII software (Campden Instruments Ltd.). Each mouse was assigned to a particular chamber for the entire duration of the study.

After completing pretraining (described by Horner et al., 2013), 5‐CSRTT training began. Each session had 40 trials, and stimulus duration was systematically reduced from 8 to 4 s, and then to 2 s. Stimulus presentation was followed by a 5‐s limited hold period when responses were still counted. Responses during the stimulus presentation or the limited hold period were registered as correct if in the location of the stimulus or incorrect if in one of the other four locations. After a response, if there was still time remaining in its presentation, the stimulus was immediately removed from the screen. If no response was made, an omission was recorded and the mouse received a 5‐s time‐out. Once the reward was collected, and following the 5‐s intertrial interval (ITI), the next trial could be initiated. After initiation and a 5‐s fixed delay period, the next trial started. If a response was made during the 5‐s delay between initiation and stimulus onset, it was recorded as a premature response and the mouse received a 5‐s time‐out. Once stimulus duration was 2 s, and mice were performing at greater than 80% accuracy and less than 20% omissions, for 3 of 4 consecutive sessions, they were moved onto 5‐CSRTT probe testing.

Probe testing sessions were identical to the 5‐CSRTT training sessions with the exception of stimulus duration, which was reduced from 2 s (baseline stimulus duration) to 1.6, 1.0, 0.8, and 0.6 s. Each stimulus duration was tested for two consecutive days, followed by 1 to 2 consecutive days of the 2‐s baseline stimulus duration to ensure stable baseline performance.

At 7‐, 12‐, and 16‐month time points, the mice were tested under baseline conditions with stimulus duration of 2 s. Once the mice were performing at >80% accuracy and < 20% omissions, for 2 consecutive sessions, they were moved onto 5‐CSRTT probe testing. There was no difference in the number of trials to criterion between genotypes during the baseline training.

At 7‐, 12‐, and 16‐month time points, four additional probes were used. The stimulus duration was reduced to 0.4 and 0.2 s, and a *Vigilance Probe* and an *Impulsivity Probe* were included. The Vigilance Probe used a 2‐s stimulus duration over 200 trials, for a maximum of 90 min. Because of the extended length of the session, the Vigilance Probe is a sensitive measure for sustained attention. The Impulsivity Probe used a 2‐s stimulus duration and 10‐s delay, instead of the 5‐s baseline delay. Because of the longer delay, the Impulsivity Probe is a sensitive measure for assessing premature responding (Dalley et al., 2007).

The number of sessions to reach the criterion performance at each stage of pretraining and 5‐CSRTT training was recorded. For the *5‐CSRTT Probes,* the following behavioral variables were evaluated: *accuracy, omissions, premature responding, perseverative responding, reward response latency, correct response latency, incorrect response latency, beam breaks front,* and *beam breaks back*. *Accuracy* was defined as percentage correct and was calculated as the number of trials in which a response was made to a correct location, divided by the total number of both correct and incorrect trials. *Omissions* were defined as the percentage of all trials (i.e., correct + incorrect + omissions) in which the animal made no response. *Premature responses* were defined as the number of touches made during the delay period prior to a stimulus appearing and was used as a measure of impulsivity. *Perseverative responding* was defined as the number of screen touches after a correct response, prior to collecting the reward, and was used as a measure of compulsivity. *Response latency* was defined as the time between a stimulus appearing on the screen and the animal making a response. *Reward response latency* was defined as the time taken to collect the reward after a correct response. Beam breaks were defined as the number of times the mouse crossed the infrared beams near the screen (i.e., *beam breaks front*) or magazine (i.e., *beam breaks back*).

Data were analyzed by converting trial data to group means on all of the performance measures described above, and analyzed using repeated measures ANOVA, with a within‐subject factor of stimulus duration and a between‐subject factor of genotype. All statistical analyses described in this manuscript were conducted with SPSS version 22 and Microsoft Excel version 14.4.5. Statistical significance was set at *p* < .05, unless running a Bonferonni post hoc comparison. All data are presented as mean ± *SEM*.

### 2.3 Object recognition

To evaluate object recognition memory, the *Decoupled* version of the OR paradigm was used (McTighe, Cowell, Winters, Bussey, & Saksida, 2010). This is a spontaneous task that does not require training and takes advantage of a rodent's natural preference toward novelty. Time spent exploring the novel and familiar objects is analyzed and used to infer memory (Ennaceur & Delacour, 1988). Thirty min prior to testing, mice were brought into a holding room that was illuminated by a red light and adjacent to the testing room. All OR testing was carried out under dim white light conditions. Mice were individually transported in a cardboard carrying box between the holding room and the testing room.

OR testing took place in a Y‐maze (previously described in Romberg et al., 2012) made of homogenous opaque white Perspex. Walls were 30‐cm high and each arm was 16 cm in length and 8‐cm wide. One arm was used as the start arm, and the other two arms were used to present the testing stimuli, which were randomly shaped objects (dimensions approximately 10 cm × 4 cm × 4 cm) secured to the floor of the maze using Blu‐tack™. The maze and objects were wiped with a 50% ethanol solution and dried between trials. The objects used and side of the maze in which the novel object was presented were counterbalanced.

Mice received two daily 5‐min sessions of habituation to the empty maze prior to the first trial of OR. At later time points (i.e., 8, 13, and 17 months of age), only 1 day of habituation for 5 min was conducted prior to testing.

Testing was divided into two phases: *sample* phase and *test* phase. During the sample phase, the mouse was placed in the start arm of the Y‐maze and allowed to explore two identical objects located at the ends of the other two arms for 5 min. Mice were then removed from the maze and placed in their home cage for either a 1‐ or 24‐hr delay period. For the test phase, mice were placed back into the same Y‐maze apparatus and presented with one of two conditions for 5 min: *repeat* condition or *novel* condition. For the repeat condition, the same two identical objects (i.e., familiar) seen during the sample phase were presented. For the novel condition, two new (i.e., novel) identical objects were presented. For each delay, mice were tested in both the repeat and novel conditions, using distinct object pairs, for a total of four trials at each time point. Trials were separated by at least 48 hr to prevent interference and to prevent declining motivation. Objects were counterbalanced between mice to control for object bias.

Exploration was defined as a mouse directing its nose to an object at a distance of 2 cm or less. Sitting on or chewing at the base of the object was not included as exploration. Exploration was scored blind to genotype and condition, using JWatcher_V1.0, written in Java[TM] (JWatcher, USA). For Decoupled OR, discrimination ratios (D2) were calculated for both the *repeat* and *novel* conditions for each time delay, and calculated as follows:

$$D2=\frac{\text{Test Phase exploration}(s)}{\text{Sample Phase exploration}(s)}$$

D2 scores < 1 on the *repeat condition* suggested that the mouse viewed the test objects as familiar and were interpreted as a subject remembering the sample objects. It was hypothesized that D2 scores would be ~1 for the *novel condition*. A D2 < 1 in the novel condition is interpreted as a false memory, such that the mouse saw the new object as familiar (McTighe et al., 2010). Sample data were compared using independent Student's *t* tests, to ensure total exploration during the sample phase was equal between the genotypes for each condition. Choice data were analyzed using a repeated measures ANOVA, with post hoc Student's *t* contrasts.

## 3 RESULTS

### 3.1 No differences between Tg^+^ and Tg^−^ on 5‐CSRTT measures of attention and executive control at 5, 7, 12, and 16 months of age

**Figure 1:**
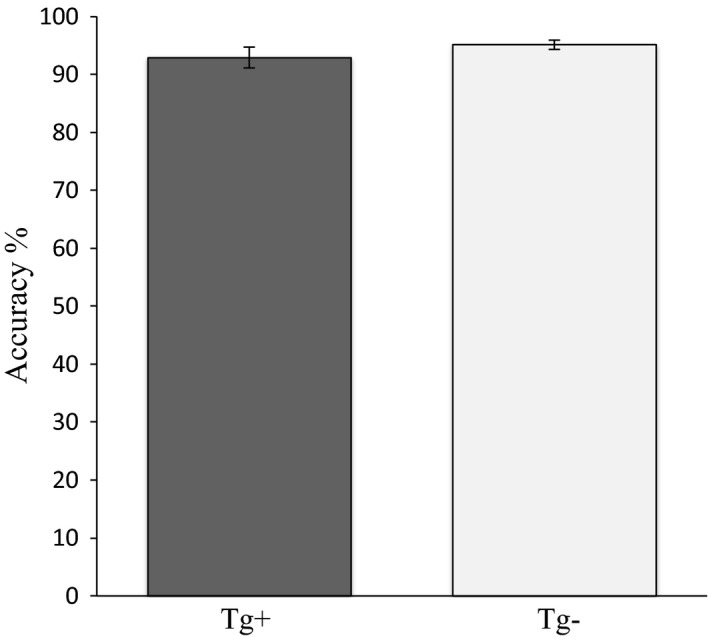
Baseline accuracy at the start of 5‐CSRTT probe trials. The *y*‐axis represents the mean accuracy (%) for the final two baseline sessions prior to the start of probes. There was no difference between Tg^+^ and Tg^−^. Data are expressed as the mean ± *SEM*

There were no differences between Tg^+^ and Tg^−^ for sessions to criterion during 5‐CSRTT *pretraining*, during baseline 5‐CSRTT performance prior to the start of probe testing, or throughout probe testing (*p *> .05; data not shown). There was also no difference between Tg^+^ and Tg^−^ baseline accuracy at the start of 5‐CSRTT probe trials (Figure 1).

**Figure 2:**
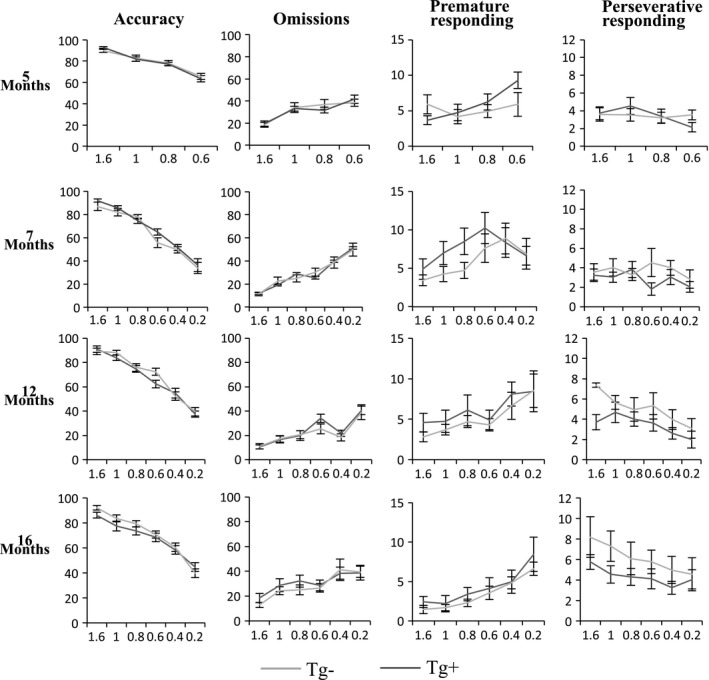
5‐CSRTT performance on probe trials using decreased stimulus duration. Line graphs showing *accuracy* (%), *omissions* (%), number of *premature responses*, and number of *perseverative responses* for Tg^+^ and Tg^−^ on 5‐CSRTT at 5, 7, 12, and 16 months of age. The *y*‐axis represents the mean performance value, and the *x*‐axis represents the stimulus duration for each probe. There were no statistically significant interactions between genotype and stimulus duration for any performance measure, at any time point (*p* > .05). Data are expressed as the mean ± *SEM*

Attention and executive control were evaluated using 5‐CSRTT at 5, 7, 12, and 16 months of age. For probes of decreasing stimulus duration (i.e., 1.6, 1.0, 0.8, 0.6, 0.4, 0.2 s), measures of performance used to compare Tg^+^ and Tg^−^ were as follows: accuracy, omissions, premature responding, perseverative responding, reward response latency, correct response latency, incorrect response latency, beam breaks front, and beam breaks back. Repeated measures ANOVAs showed no statistically significant interactions between genotype and stimulus duration on any of the performance measures, at any of the time points. Figure 2 provides line graphs illustrating *accuracy*,* omissions, premature responses*, and *perseverative responses* at 5, 7, 12, and 16 months of age. Each probe was run for two consecutive days, so each data point is an average of the two days (data for other measures not shown).

**Figure 3:**
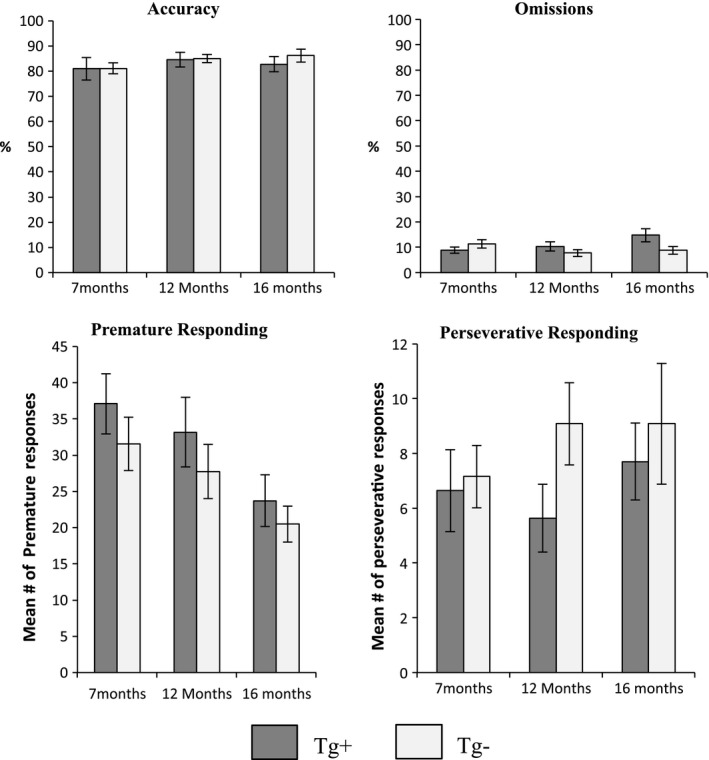
5‐CSRTT Impulsivity Probe. *Accuracy* (%)*, omissions* (%)*,* mean number of *premature responses,* and mean number of *perseverative responses* for Tg^+^ and Tg^−^ during the 5‐CSRTT Impulsivity Probe with a 10‐s delay. Bar graphs show the performance measures at 3 time points: 7, 12, and 16 months of age. There were no statistically significant differences in accuracy between Tg^+^ and Tg^−^ at any of the time points. Omission rate at 16 months of age is higher for Tg^+^ than Tg^−^ but not statistically different (*p *= .052). Premature responses decrease with age, which may be a result of learning (main effect of age *p* = .04). No statistically significant differences in perseverative responding between Tg^+^ and Tg^−^ were detected. At 12 months of age, Tg‐ make more perseverative responses but it is not statistically significant (*p =* .09). Data are expressed as the mean ± *SEM*

**Figure 4:**
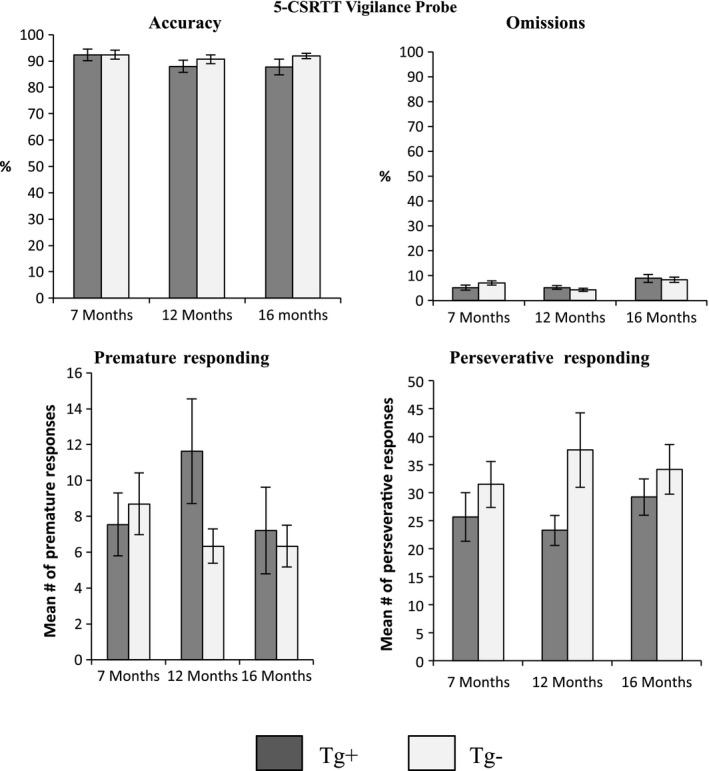
5‐CSRTT Vigilance probe. *Accuracy* (%)*, omissions* (%)*,* mean number of *premature responses,* and mean number of *perseverative responses* for Tg^+^ and Tg^−^ during the 5‐CSRTT *Vigilance probe* with 200 trials. There were no statistically significant differences on any of the measures, at any of the time points. At 12 months of age, Tg^+^ show more premature responses than Tg^−^ (*p =* .09) and Tg‐ show more perseverative responses than Tg^+^ (*p =* .07). Data are expressed as the mean ± *SEM*

Similarly, there were no statistically significant effects on any of these performance measures for the *Impulsivity Probe* (10 s delay; Figure 3) or *Vigilance Probe* (200 trials; Figure 4) tested at 7, 12, and 16 months of age (*p* > .05).

### 3.2 No differences between Tg^+^ and Tg^−^ on decoupled OR with 1‐ or 24‐hr delays at 6, 8, 13, or 17 months of age

**Figure 5:**
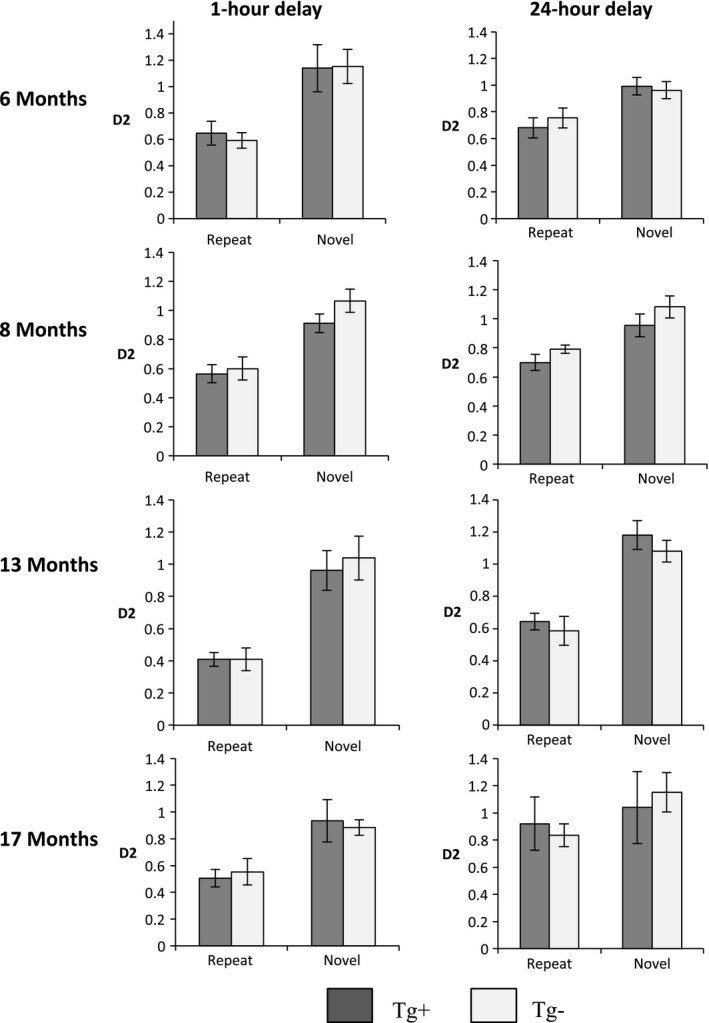
Decoupled OR at 6, 8, 13, and 17 months of age. Bar graphs showing D2 on the *y*‐axis, comparing Tg^+^ and Tg^−^ on *Repeat* and *Novel* conditions. There were no statistically significant differences between Tg^+^ and Tg^−^ at any time point. Data are expressed as the mean ± *SEM*

Mice were tested on Decoupled OR with a 1‐ and 24‐hr delay at 6, 8, 13, and 17 months of age. There were no statistically significant differences in sample exploration between Tg^+^ and Tg^−^ at any time point (data not shown). Repeated measures ANOVAs revealed no statistically significant interactions (*p *> .05) between D2 scores of Tg^+^ and Tg^−^ on *Repeat* or *Novel* conditions at 6, 8, 13, and 17 months of age (Figure 5).

## 4 DISCUSSION

This study provides the first longitudinal cognitive profile of the TgTau^P301L^ mouse, from 5 to 17 months of age, using behavioral tasks to evaluate attention, executive functioning, and object recognition memory. Frontal cortex‐dependent executive function and attention were evaluated using the touchscreen version of the 5‐CSRTT at 4, 7, 12, and 16 months of age. Object recognition memory was assessed at 5, 8, 13, and 17 months of age. Because the TgTau^P301L^ mouse model develops tau pathology slowly, there may have been sufficient time for compensatory changes to mask some of the phenotypes (e.g., attentional impairments) typically seen in patients with dementia. We conclude that this model is not ideal for studying early impairments in attention, executive functioning, or recognition memory. However, spatial and object recognition memory impairments were observed during follow‐up assessments when the mice were 18 and 21 months, respectively (see Appendix for the Supplementary methods and results). These impairments are consistent with previous publications, and with a dementia‐like phenotype in these mice when aged.

P301L is the tau mutation most frequently observed in patients with FTDP‐17 (Poorkaj et al., 2001). The TgTau^P301L^ transgenic mouse model has been previously shown to recapitulate the progressive development of glial fibrillary (GFT) and NFT, cerebral atrophy, and age‐related cognitive impairments observed in patients (Murakami et al., 2006; Sasaki et al., 2008; Wakasaya et al., 2011). For example, Sasaki et al. (2008) compared immunocytochemical analyses of brains from six patients with tauopathies (including AD) and TgTau^P301L^ mice at 11–27 months of age. The TgTau^P301L^ mice showed microglial activation in gray matter associated with phosphorylated tau deposition, which was similar to samples from the human patients. There are also many similar factors responsible for NFT formation and neuronal cell loss between the TgTau^P301L^ mice and both patients with AD and FTD, as demonstrated by comparing oligonucleotide array expression (Wakasaya et al., 2011). These comparative studies validated the TgTau^P301L^ mice as a model of tauopathies, including both FTD and AD.

The TgTau^P301L^ model was first characterized by Murakami et al. (2006), who reported initial tau pathology development in the hippocampus, amygdala, and cerebral cortex at approximately 3 months of age and tau‐positive pretangles at 10 months of age. Although not specified explicitly in the histological report for this cohort at 3 or 10 months of age, we assumed that “cerebral cortex” was referring to the frontotemporal cortex because that was the site of the most extensive pathological markers at later ages. For example, extensive NFTs were identified throughout the frontotemporal cortex at 18 to 24 months of age. Histological analysis of another cohort of TgTau^P301L^ at 13 months of age showed that 37% had pretangles, 42% had pretangles and GFTs, and 21% had pretangles, GFTs, and NFTs. NFTs were found in the cerebral cortex, hippocampus, amygdala, basal forebrain nucleus, locus ceruleus, and substantia nigra. Glial tau pathology developed independently and preceded neuronal cytopathology. Mice showed brain atrophy by 18 months of age, in the temporal cortex, including hippocampus. Tau‐positive glial tangles were also observed in the spinal cord. These histological reports of the TgTau^P301L^ model illuminate the high within‐cohort variance in pathology. The authors suggest that this variance may be caused by genetic modifiers, environmental parameters, or stress (Murakami et al., 2006). The high variability in pathology may explain the variability in behavior demonstrated by our cohort.

Because of the regional specificity of tau pathology in the TgTau^P301L^ model, the present study prioritized tasks dependent upon the frontotemporal cortex. The cohort was first tested on 5‐CSRTT because evidence suggests that executive and attentional deficits may be the earliest cognitive deficits in AD, prior to deficits in spatial memory and language impairments (Baddeley et al., 2001; Collette et al., 1999; Lawrence & Sahakian, 1995; Perry et al., 2000), and may be a predictive preclinical feature of AD (Albert et al., 2001). Given the importance of early detection and the slow progression of pathology in this model, we thought that examining executive and attentional deficits provided the best chance at detecting the earliest cognitive changes.

To our knowledge, only limited behavioral characterization of these mice has been performed, which did not investigate the earliest cognitive changes. Murakami et al. (2006) evaluated the TgTau^P301L^ mouse model on the Morris Water Maze (MWM) at 9 and 12 months of age and the eight‐arm radial maze at 9 and 13 months of age. Older cohorts were tested on the open‐field test, MWM (reference memory and visible cued platform test), and conditioned taste aversion. The results showed impaired working memory at 12 and 13 months of age and impaired conditioned taste aversion at 16–18 months of age. Importantly, unlike the present study, these data were collected from a cross‐sectional rather than longitudinal design.

The present longitudinal study evaluated several cognitive domains. Firstly, using a touchscreen version of the 5‐CSRTT, we examined frontal cortex‐dependent executive function and attention at 4, 7, 12, and 16 months of age in the TgTau^P301L^ mice. By comparing these data to the results of studies of other rodent models of dementia using the same testing method, an interesting profile of behavioral differences emerges, which may be related to the precise pathological insult experienced. Specifically, Romberg et al. (2011) tested attention and executive control in 3xTgAD mice, which express the APPswe, and PS1 M146V mutations in concert with the tau P301L mutation. Subsequently, the TgCRND8 mouse, a widely used model of Aβ pathology, expressing the Appswe/ind mutation was also evaluated in this paradigm (Romberg et al., 2013). The 3xTgAD model was found to perform with less accuracy and make more perseverative responses than the control mice at 9 months of age in this task. In contrast, 4‐ to 5‐month‐old TgCRND8 mice exhibited lower accuracy, but no differences in other measures including perseverative responding. Our results now add to this profile, reporting that expression of the tau P301L mutation alone has no effect on touchscreen 5‐CSRTT performance across a wide range of ages.

Secondly, we examined the TgTau^P301L^ mouse model using the Decoupled OR task. Task performance was unaffected at 5, 8, 13, and 17. This suggests a remarkable functional resilience of medial temporal cortical structures, given the likely extensive nature of the pathological insult experienced by the 17‐month time point. As with the 5‐CSRTT assessment, we have previously examined the performance of the TgCRND8 amyloid model in the Decoupled OR paradigm. Romberg et al. (2012) found that the Tg^+^ did not perform differently on the repeat and novel conditions in the Decoupled OR task, whereas the littermate controls showed higher D2 scores in the novel condition. This was interpreted as the TgCRND8 exhibiting recognition memory impairment due to false recognition rather than forgetting. False recognition has been reported as a cause of memory impairments in patients with AD and those with MCI (Abe et al., 2011; Budson, Desikan, Daffner, & Schacter, 2001; Gold, Marchant, Koutstaal, Schacter, & Budson, 2007; Hart, Smith, & Swash, 1985; Hildebrandt, Haldenwanger, & Eling, 2009; Plancher, Guyard, Nicolas, & Piolino, 2009; Yeung, Ryan, Cowell, & Barense, 2013). Unlike the TgCRND8 model, no memory impairment was observed in the TgTau^P301L^ model.

Following the completion of the longitudinal study and the strikingly similar performance of the Tg^+^ and Tg^−^ groups, we decided to conduct further cognitive tasks and histological analysis. The methods and results can be found in the Appendix (Table S1). We assessed spatial memory using the hippocampus‐dependent location recognition (LR) and T‐maze tasks between 18 and 20 months of age, and recognition memory using the Forced‐choice version of OR at 19 and 21 months of age. Spatial memory was assessed because previous studies had demonstrated impairments in this mouse model on the MWM and radial arm maze between 9 and 13 months of age (Murakami et al., 2006). The Forced‐choice paradigm was chosen because it requires half as many trials as the Decoupled paradigm, which makes it faster to assess different delays. At 17 months of age, performance at the 24‐hr delay on the repeat condition of the Decoupled version was approaching a discrimination ratio of 1, which suggested that both groups were having trouble remembering at such a long delay. The Forced‐choice paradigm enabled us to evaluate intermediate delays (i.e., 3 and 8 hr). As the mice were becoming aged, and the attrition rate was increasing, it was important to test different delays as quickly as possible.

On the LR task, the results were variable but suggestive of mild spatial memory impairment in the TgTau^P301L^ mice at 18 months of age (Figure S1). This observation is consistent with the hippocampal atrophy observed in mice of the same age. Due to high levels of subcriterion performance, it was not possible to draw conclusions from the data collected using the T‐maze task (Figure S2). Considering the LR deficit observed here and the fact that spatial deficits have been reported in these mice at younger ages (Murakami et al., 2006), future studies of this model should prioritize early detection of hippocampus‐dependent deficits. For example, there is some evidence that mice expressing the P301L transgene are particularly impaired in trace fear conditioning relative to other hippocampal‐dependent tasks such as the MWM (Hunsberger et al., 2014); thus, researchers using TgTau^P301^ mice might consider employing trace fear conditioning tasks to detect the earliest memory deficits.

On the Forced‐choice OR paradigm, the TgTau^P301L^ mice exhibited a deficit at 21 months, but not at 19 months of age (Figure S3). However, due to the limitations of the Forced‐choice technique, it is not possible to determine whether the deficit in the TgTau^P301L^ animals is due to false memory or forgetting. Future studies could evaluate TgTau^P301L^ at 21 months of age on the Decoupled version of OR to confirm the nature of this deficit and enable comparison with the previous TgCRND8 study (Romberg et al., 2013). The decision to terminate the behavioral testing at 21 months of age was because of the uneven sample sizes, the increasing attrition rate of the TgTau^P301L^ sample, and the need to conduct histological analysis to confirm tau pathology in the Tg^+^ group and the absence in Tg^−^ group (Figures S4 and S5). Future studies should use larger sample sizes to investigate the specific impairments at such late time points.

The greatest strength of the longitudinal design is that we were able to control for cohort effects while identifying changes as pathology develops. Cohort effects are particularly relevant to transgenic models, which can be affected by genetic drift in the colony. Differences between cohorts caused by factors other than genotype and pathology are not easily ruled out when comparing cohorts of different ages. The repeated testing also allows for the timing of the onset of cognitive impairments to be detected.

A potential issue with our longitudinal study would be the possibility of contamination by repeated exposure to the tasks. This is particularly a concern for repeated testing on the 5‐CSRTT because training and learning are required. It is possible that learning from the repeated testing could have masked differences between the genotypes. However, this is unlikely because the mice displayed similar duration‐dependent performance at various ages, such that, as the duration of the stimuli was shortened, performance declined. Additionally, we were comparing between genotypes and both groups received the same amount of training. Contamination caused by repeated testing is not likely an issue for the spontaneous tasks (e.g., OR) because no training or learning is required. There is no reason to expect that repeated exposure to the testing chamber would affect novelty preference during OR, particularly because all mice were habituated to the chamber prior to the initial tests.

In summary, this study represents the first longitudinal behavioral evaluation of the TgTau^P301L^ mouse model of tauopathy. There were no apparent changes in executive function or attention in these animals as measured in the touchscreen 5‐CSRTT. However, spatial and object recognition memory impairments were observed during follow‐up assessments using LR and Forced‐choice OR tasks when the mice were 18 and 21 months, respectively. These impairments are consistent with a dementia‐like phenotype in these mice when aged. Thus, despite using tasks proven to be sensitive with mouse models of neurodegenerative disease (Romberg et al., 2011, 2013), no behavioral impairments were identified until the mice were aged. It seems unlikely that this was due to an absence of pathology prior to 18–21 months of age, as Murakami et al. (2006) showed pretangles, GFTs, and NFTs by 13 months of age, and we observed significant pathology at our final time point. To conclude, this model may be useful for studying impairments in some aspects of cognitive function relevant to neurodegenerative disease, at later stages of disease. The model may not be as useful for research aiming to detect early changes in attention, executive function, and recognition memory.

## DISCLOSURE

LMS and TJB consult for Campden Instruments Ltd.
