# Supplementary material for: Longitudinal evaluation of Tau‐P301L transgenic mice reveals no cognitive impairments at 17 months of age
Source: Brain Behav. 2017 Dec 18;8(1):e00896. doi: 10.1002/brb3.896 (PMC5853624; doi:10.1002/brb3.896)
Supplement: Supplementary file 1 [file BRB3-8-e00896-s001.docx]

**Appendix**

**Supplementary Methods:**

**Table S1. Timeline of additional testing and sample sizes**

| **Age (months)** | **Behavioral Task** | **Sample Size** |
| --- | --- | --- |
| **18** | **Location Recognition (1 h delay)** | **N = 21**  **(9 Tg^+^ and 12 Tg^-^)** |
| **19** | **Forced-choice OR (3 h and 8 h delay)** | **N = 19**  **(7 Tg^+^ and 12 Tg^-^)** |
| **20** | **T-maze** | **N = 18**  **(6 Tg^+^ and 12 Tg^-^)** |
| **21** | **Forced-choice OR (8 h delay)** | **N = 17**  **(6 Tg^+^ and 11 Tg^-^)** |
| **22** | **Testing complete. Histology performed.** | **N = 17**  **(6 Tg^+^ and 11 Tg^-^)** |

***Forced-choice OR*:** Testing consisted of two phases: *sample* phase and *test* phase. The 5 min sample phase was identical to Decoupled OR described above. However, during the test phase, mice were presented with one copy of the object used previously during the sample phase (i.e., *familiar* object) and a new object (i.e., *novel* object). Mice were allowed to explore the maze and objects for 5 min. Forced-choice OR used 3 h or 8 h delays between the sample and test phases, in which the mouse was returned to its home cage.

For *Forced-choice OR,* preference for the novel object was calculated as:

$$D2=\frac{novel object exploration- familiar object exploration\left( s \right)}{novel object exploration+ familiar object exploration (s)}$$

D2 scores > 0 represent a novelty preference. Sample data was compared using independent Student’s *t*-tests, to ensure total exploration during the sample phase was equal between the genotypes for each condition. Choice data from Forced-choice OR was analyzed using independent Student’s *t*-tests comparing genotype in each condition.

***Location Recognition (LR):*** To evaluate spatial memory we used the LR task (Warburton et al., 2000). Much like OR described above, LR takes advantage of a rodent’s innate preference towards novelty so no training is required. The testing room was dimly lit with white light and had distinct distal and proximal spatial cues.

LR testing took place in a black plastic circular arena (43 cm diameter, 17 cm tall walls) with 1 cm of bedding on the floor. A digital video camera was mounted above the maze to record all trials. The stimuli were randomly shaped objects (dimensions approximately 10 cm x 4 cm x 4 cm). All objects presented were new and had not been seen previously. The objects were wiped with a 50 % ethanol solution and dried between trials and secured to the floor of the maze using Blu-tack ^TM^.

Each mouse was given 4 consecutive daily sessions of habituation to the maze, where they were allowed to freely explore the empty maze for 5 min. Each trial consisted of two phases: *sample* phase and *test* phase*.* During the sample phase, each mouse was given 5 min to explore the arena and was shown two identical objects spaced 20 cm apart. After a 1 h delay, the mice were returned to the arena for the test phase and allowed to explore for 5 min. For the test phase mice were presented with the same two identical objects previously used during the sample phase: one in its previous (i.e., familiar) location and one in a new (i.e., novel) location. The novel location was always directly across from the object in the familiar location.

Exploration was defined and scored as OR above. D2 scores were calculated as follows:

| $D2=\frac{novel location exploration- familiar location exploration\left( s \right)}{novel location exploration+ familiar location exploration (s)}$ |  |
| --- | --- |

Data was analyzed using independent Student’s *t*-test.

***T-Maze:*** To evaluate spatial memory using an additional task, mice were tested using the hippocampal-dependent T-Maze (Sigurdsson et al., 2010). Testing took place in a T- shaped three-arm maze made of Perspex. Each arm was 30 cm long, 10 cm wide, and 20 cm high. The maze had a white floor and black walls, and was placed on a table 43 cm above the floor in a room lit with white light and prominent distal visual cues.

Mice received two days of habituation to the maze. During habituation, all three arms were baited with a single sucrose reward pellet (14 mg, Sandown Scientific, Middlesex, UK). The mouse was placed in the start arm with the door lowered until the pellet was consumed. The door was then removed and the animal was free to explore the maze. Once the animal had consumed the two remaining pellets, the start-arm was re-baited. When the animal had consumed the pellet in the start arm, the two choice arms were re-baited. This continued for 10 min.

Mice received two days of shaping using one open choice arm while the second choice arm was blocked. The animal was placed in the baited start arm with the door lowered. Once the mouse had consumed the pellet, the start door was removed and the mouse was allowed to explore only one of the choice arms. Once the animal returned to the re-baited start arm, the door was lowered and the alternate choice arm was baited and opened for the next trial. Each session had 10 trials with the order of the open-baited arm presented in a pseudorandom order.

Acquisition training on delayed non-match to location began on the fourth day. Training consisted of a *sample* phase and a *choice* phase*.* For the sample phase, the mouse was placed in the baited start arm, with the door lowered.  Once the pellet was consumed, the start arm was opened and the mouse was allowed to enter only one of the choice arms. Once the animal consumed the pellet in the choice arm, the start arm was re-baited. When the animal returned to the start arm, the start door was closed, and the maze was wiped with a 50% ethanol solution and the other choice arm door was opened. After a 10 s delay, the choice phase began. The start arm door was opened and the mouse could choose to enter either the left or right choice arms. The *correct* choice was the arm not previously visited during the sample phase. Once the mouse entered a choice arm, the opposite arm was immediately closed. The start arm was re-baited and the next trial began once the mouse returned to the start arm. Each daily session had 10 trials. Testing was conducted for 24 consecutive days.

An accuracy score for each daily session was calculated as the number of correct trials out of 10. Data was analyzed using a repeated measures ANOVA comparing genotype across trials. The number of trials to criterion was also calculated. Criterion was an accuracy score of 70 % for three consecutive days. Independent samples Student’s *t*-test was used to analyze trials to criterion.

**Histology:** At the end of the experiment, mice were anaesthetized by intraperitoneal (IP) injection of Dolethal (0.3 ml; Vetoquinol UK Ltd., Buckinghamshire, UK) and perfused transcardially with phosphate buffered saline (PBS) for 2 min, followed by 4% neutral buffered formalin (NBF) for 5 min. Brains were removed and post-fixed in NBF for at least 24 h at 4 °C, followed by immersion in 70 % ethanol at 4 °C.

Half brains of Tg+ and Tg- mice were dissected into 10% buffered formalin and kept at 4^o^C overnight. Samples were then embedded in paraffin and sagittal sections (10 µm) were probed with the AT8 monoclonal antibody (1:400, Innogenetics) which recognizes phosphorylated PHF-tau (serine-202/threonine-205). An HrP-conjugated anti-mouse IgG (Sigma) was used as a secondary antibody and sections were counterstained with hematoxylin and eosin.

**Supplementary Results:**

***Tg+ show spatial memory impairment on LR at 18 months of age***

At 18 months of age, mice were tested on the LR task using a 1 h delay. Due to highly variable performance, each mouse was tested on 4 trials. An independent Student’s *t*-test comparing the average D2 scores across the four trials revealed that the Tg+ showed less of a preference for the novel location than the Tg- group (*p =* 0.018). Figure S1 shows combined data across the four trials because of the high variability in performance. There were no differences in sample exploration (*p* > 0.05).

***Tg+ show recognition memory impairment on Forced-choice OR with an 8 h delay that develops between 19 and 21 months of age***

Mice were tested on Forced-choice OR at 19 months of age using a 3 h and 8 h delay. There was no statistically significant difference in D2 scores between Tg+ (M= 0.20; SEM= 0.063) and Tg- (M= 0.20; SEM= 0.086) after a 3 h delay. Three trials of the 8 h delay were run because of high variability; however, there was no statistically significant difference in D2 scores between Tg+ and Tg- after the 8 h delay at 19 months of age. At 21 months of age, mice were re-tested with two trials of Forced-choice OR with an 8 h delay. The Tg+ group showed no preference for the novel object, and significantly less preference for the novel object than the Tg- group (*p* = 0.022), suggesting that Tg+ had impaired recognition memory at 21 months of age. Figure S2 shows the D2 scores at 19 and 21 months of age for Forced-choice OR with an 8 h delay.

***Tg+ and Tg- show highly variable and inconclusive spatial memory performance on T-Maze at 20 months of age***

Tg+ and Tg- were tested on the T-maze for 24 consecutive days. The mice showed highly variable and inconclusive performance. Criterion was an accuracy score of 70 % for three consecutive days, but only 4 Tg+ and 10 Tg- reached the performance criterion. Of these, there was no statistically significant difference between the number of trials Tg+ (M = 6.0; SEM = 1.47) and Tg- (M = 9.1; SEM = 1.30) took to reach criterion. Because of the low and highly variable performance, all mice were maintained on the 10 s delay and never tested on probe trials with longer delays. On the baseline performance, there was no statistically significant interaction between genotype and block or main effect of genotype on performance accuracy. Figure S3 presents the performance data in blocks of three consecutive days.

***Tg+ but not Tg- show extensive tau pathology throughout the frontotemporal cortex at 22 months of age***

Histological analyses identified extensive tau pathology throughout the frontotemporal cortex in the Tg+, but not Tg-. Figure S4 shows representative images of Tg+ and Tg- sagittal sections. Figure S5 shows representative images of intracellular tau inclusions.

**Supplementary Figure Legends:**

**Figure S1.** LR with 1 h delay at 18 months of age. Y-axis shows the average D2 scores for Tg+ (left) and Tg- (right) after a 1 h delay at 18 months of age. Tg+ showed less of a preference for the novel location (i.e., lower D2 score) than the Tg- group (*p =* 0.018). Means and ± SEM shown.

**Figure S2.** Forced-choice OR with 8 h delay. Bar graphs showing D2 scores on the y-axis for Tg+ and Tg- at 19 and 21 months of age. There was no difference between D2 scores at 19 months of age; however, at 21 months of age, the Tg+ group showed significantly less preference for the novel object than the Tg- group (*p* = 0.022). Data are expressed as the mean ± SEM and combined data from three trials at 19 months of age and two trials at 21 months of age.

**Figure S3.** T-maze task at 20 months of age. No statistically significant effect of genotype on performance was detected. Each block consisted of three consecutive daily sessions (10 trials per session). The y-axis shows the mean number of correct trials (out of 10). Chance performance is a score of 5 and illustrated by the dotted line. Data are expressed as the mean ± SEM.

**Figure S4.** Representative sagittal sections from Tg+ and Tg- revealing extensive tau pathology throughout the frontotemporal cortex of the Tg+ (top), but not in Tg- (bottom).

**Figure S5.** Representative images showing intracellular tau inclusions in the frontal cortex (left) and hippocampus (right) in a Tg+ mouse.

**Figure S6.** 5-CSRTT pretraining sessions to criterion. The y-axis is the mean number of sessions during pretraining that it took each group to reach criterion performance and move onto probe trials. There was no difference between Tg+ and Tg-. Data are expressed as the mean ± SEM.

**Figure S7.** 5-CSRTT probe trial response latency at 5 months of age (A), 7 months of age (B), 12 months of age (C), and 16 months of age (D). The y-axis is the mean response time for correct trials. There was no difference between Tg+ and Tg-. Data are expressed as the mean ± SEM.

**Figure S8.** Total exploration time during the decoupled object recognition task at 6 months of age (A), 8 months of age (B), 13 months of age (C), and 17 months of age (D) for the 1 h delay condition (left) and the 24 h delay condition (right). The y-axis is the total time spent exploring the objects. There was no difference between Tg+ and Tg-. Data are expressed as the mean ± SEM.

**Supplementary Figures:**

**Figure S1.**


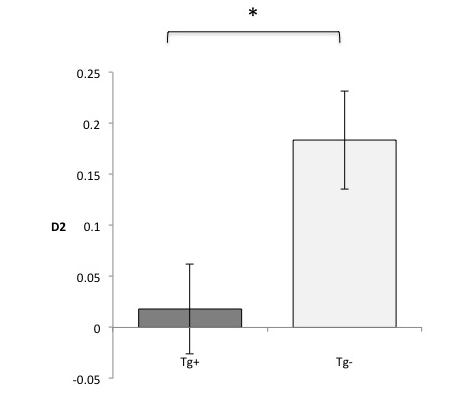


**Figure S2.**


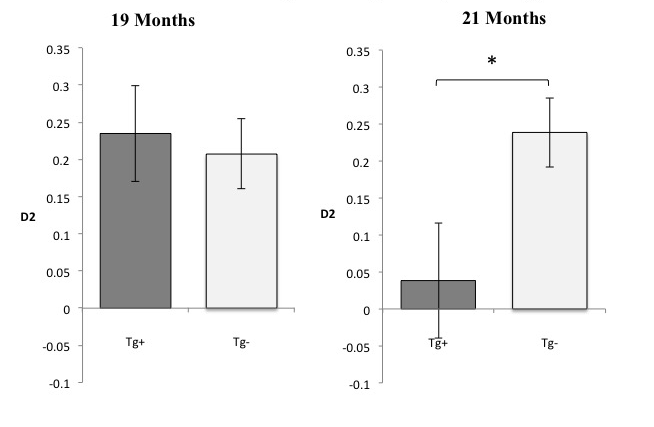


**Figure S3.**


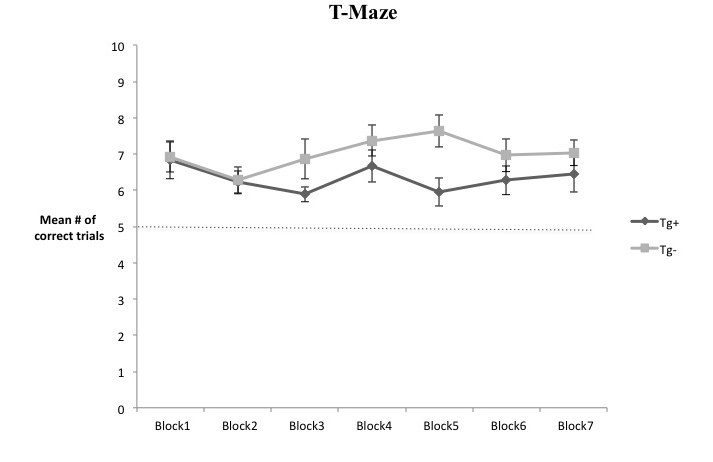


**Figure S4.**

**
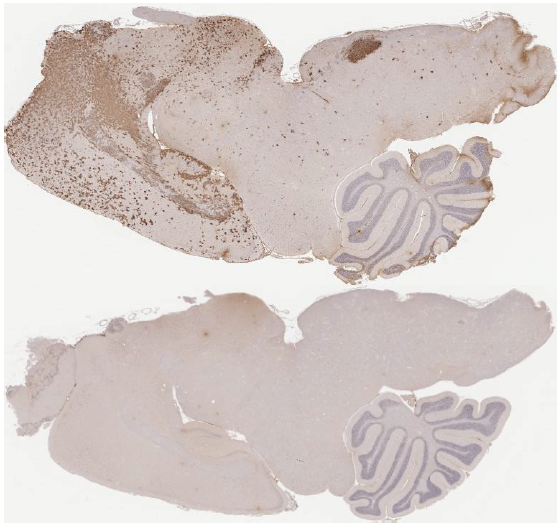
**

**Figure S5.**

***
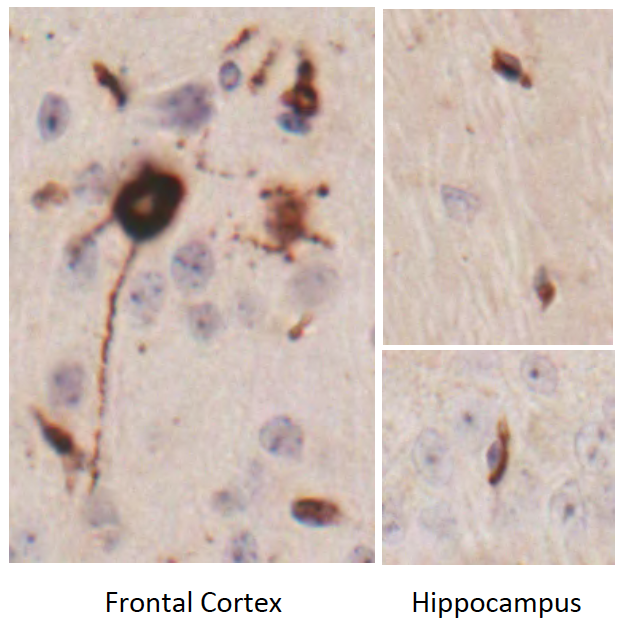
***

**Figure S6.**


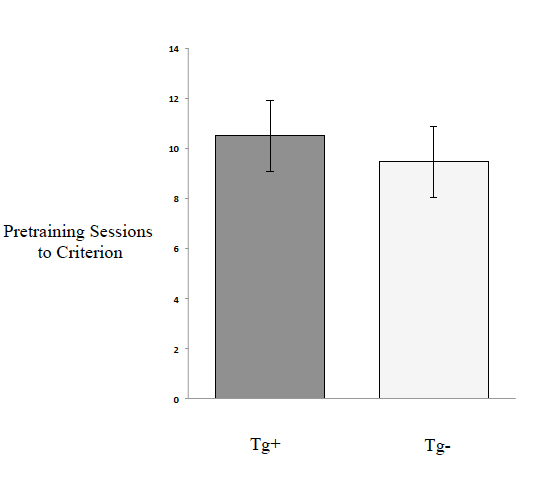


**Figure S7.**

**A)**

**B)**

**C)**

**D)**

**Figure S8**.

**A)**

**B)**

**C)**

**D)**
